# Efficacy of Caries Removal by Carie-Care and Erbium-doped Yttrium Aluminum Garnet Laser in Primary Molars: A Scanning Electron Microscope Study

**DOI:** 10.5005/jp-journals-10005-1533

**Published:** 2018-08-01

**Authors:** Attiguppe Prabhakar, Manjunath Lokeshwari, Saraswathi V Naik, Chandrashekar Yavagal

**Affiliations:** 1 Professor and Head, Department of Pedodontics and Preventive Dentistry, Bapuji Dental College & Hospital, Davangere, Karnataka, India; 2 Postgraduate Student, Department of Pedodontics and Preventive Dentistry, Bapuji Dental College & Hospital, Davangere, Karnataka, India; 3 Reader, Department of Pedodontics and Preventive Dentistry, Bapuji Dental College & Hospital, Davangere, Karnataka, India; 4 Professor, Department of Pedodontics and Preventive Dentistry, Maratha Mandal’s Nathajirao G. Halgekar Institute of Dental Sciences & Research Centre, Belgaum, Karnataka, India

**Keywords:** Carie-Care, Caries excavation, Carious primary molars, Erbium-doped yttrium aluminum garnet laser, Light microscopy, Round tungsten carbide bur, Scanning electron microscope.

## Abstract

**Aim:**

To compare and evaluate morphological changes and bacterial deposits in primary carious molars after caries excavation with Carie-Care, erbium-doped yttrium aluminum garnet (Er:YAG) laser, and round tungsten carbide bur.

**Materials and methods:**

Thirty human carious primary molars extracted for therapeutic reasons were sectioned mesiodistally. These sectioned samples were allocated into three groups (20 samples each): group I: Carie Care, group II: Er:YAG laser, and group III: round tungsten carbide bur. After caries excavation, all samples were processed and examined under conventional light microscope to examine for bacterial deposits. Representative samples from each group were processed and analyzed to examine the morphology of caries-excavated tissue by scanning electron microscope (SEM). Statistical analysis was done using Fisher’s exact test, Kruskal–Wallis test, and Mann-Whitney U test.

**Results:**

The Er:YAG laser showed best results with no smear layer followed by chemomechanically excavated surfaces with Carie-Care. Amount of bacterial deposits was observed to be more in group I while least in group II (p-value < 0.001). Mann-Whitney U test and Fisher’s exact test revealed that there was statistically significant difference among all the three groups.

**Conclusion:**

Among the three different methods of caries excavation, Er:YAG laser was found to be more effective compared with Carie-Care and round tungsten carbide bur.

**Clinical significance:**

Laser-induced caries excavation by Er:YAG laser and chemomechanical method of caries removal by Carie-Care can be considered as future of noninvasive pediatric and preventive dentistry.

**How to cite this article:** Prabhakar A, Lokeshwari M, Naik SV, Yavagal C. Efficacy of Caries Removal by Carie-Care and Erbium-doped Yttrium Aluminum Garnet Laser in Primary Molars: A Scanning Electron Microscope Study. Int J Clin Pediatr Dent 2018;11(4):323-329.

## INTRODUCTION

Dental caries is ubiquitous in all the population from the very beginning and has encompassed every part of the globe, thus justifying the wide spread of this pandemic disease since time immemorial. Thus, meticulous management of carious teeth is a common yet significant challenge faced by clinicians routinely.^1^

Historically, caries was managed by dentistry through a surgical restorative approach due to inadequate understanding of the actual disease process. This approach led to several replacement procedures, resulting in an increased restoration size or more invasive procedures over the lifetime of the given tooth.^2,3^

The search for a more gentle, comfortable, and minimally invasive technique for caries excavation has led to the development of alternative techniques, such as air abrasion, ultrasonic instrumentation, chemomechanical techniques and most recently, laser technology.^4^

Chemomechanical caries removal technique is an alternative approach to traditional caries removal that involves the use of chemical products to soften carious dentin followed by its removal with gentle excavation. One such recently introduced material is “Carie Care” gel which is an enzyme-based product aiding in non-traumatic caries removal. The presence of natural anti-inflammatory (papain gel) and clove oil (analgesic agent) compounds provide an added advantage to Carie-Care over Carisolv and Papacarie.^5^

Erbium-doped yttrium aluminum-garnet laser is considered to be least traumatic and most suited wavelength (2,940 mm) for children because of its superficial range of action coupled with zero vibration and lesser need for local anesthesia. These characteristics make Er:YAG laser an ideal option for caries removal in pediatric restorative dentistry.^6^

With the advent of modern-day minimally invasive adhesive dentistry, the morphological changes in the underlying dentin and the presence or absence of a smear layer critically affect the bonding phenomenon. Thus, the recognition of the role played by the smear layer as well as accompanying morphological changes highlight the importance of the type of cavity preparation method employed.^7^

Fusayama (1966) stated that “cariogenic bacteria were never found beyond the softened front of dentin.”^7^ Removal of infected dentin and preserving the residual affected dentin are thus prerequisites for effectively arresting the carious process without harming the long-term survival of teeth. Therefore, in defining the efficacy of some newer methods of excavation, concurrent studies on the bacteriological content of the residual dentin are of utmost importance.^8^

All the above-cited advantages and studies notwithstanding, the added benefit of minimally invasive nature, inherent safety, and effective caries excavation make Er:YAG laser and Carie-Care an even more attractive preposition for use in pediatric dentistry. However, there is a gross paucity of scientific evidence on the caries removal efficacy using histological studies and SEM in primary teeth.

Thus, the present study was contemplated to assess the morphological changes and to evaluate the efficacy in reducing the bacterial deposits in primary dentin after employing three different types of caries excavation techniques, viz., chemomechanical caries removal (Carie-Care), Er:YAG laser, and round tungsten carbide bur.

## MATERIALS AND METHODS

The present *in vitro* study was carried out in the Department of Pedodontics and Preventive Dentistry after ethical approval by Research Development and Sustenance Committee.

### Sampling Procedure

Based on previous studies, the sample size determined was 20 samples per group. Thus, 30 human carious primary molars extracted for therapeutic reasons were selected for this study. Teeth were cleaned of debris and kept in 0.9% saline solution as preservative until use. Teeth were visually examined and only primary molars with carious lesions extending up to dentin were included in the study, while teeth with gross pulpal pathology, multisurface carious lesions, developmental anomalies, or any evidence of cracks, flaws, or any damage during extraction were excluded from the study.

### Sample Preparation

The teeth were then sectioned mesiodistally in the center of carious lesion using water-cooled diamond disk, thus resulting in two samples from each tooth. Sixty samples thus obtained were stored in 0.9% saline.

### Study Groups

The samples were then randomly allocated into three groups of 20 each.

Group I: 20 carious samples excavated by Carie-Care

Group II: 20 carious samples excavated by Er:YAG laser

Group III: 20 carious samples excavated by round tungsten carbide bur

Group I

Caries excavation was done using Carie-Care gel as per the manufacturer’s instructions. It was applied for 60 seconds onto the carious surfaces after which the cavity was washed and gentle excavation was done using sharp sterile spoon excavator (1 mm). This procedure was repeated until a hard cavity surface was reached and the gel no longer remained contaminated with debris.

Group II

Carious excavation was done with Er:YAG laser (Light-Touch Laser Unit, Syneron Medical Equipment, Israel) emitting a wavelength of 2940 nm, pulse energy of 200 mJ, spot size of 1.0mm, energy density of 25.5 J/cm^2^ for 10 pps (pulse per second), in noncontact mode with water cooling. Excavation was stopped when hard dentin was detected with straight blunt probe.

Group III

Carious excavation was done with airotor using round tungsten carbide bur. Spoon excavator was used to remove soft carious tissue. Excavation was terminated when hard dentin was detected using straight blunt probe.

## EVALUATION OF BACTERIAL COUNTS

**Fig. 1:**
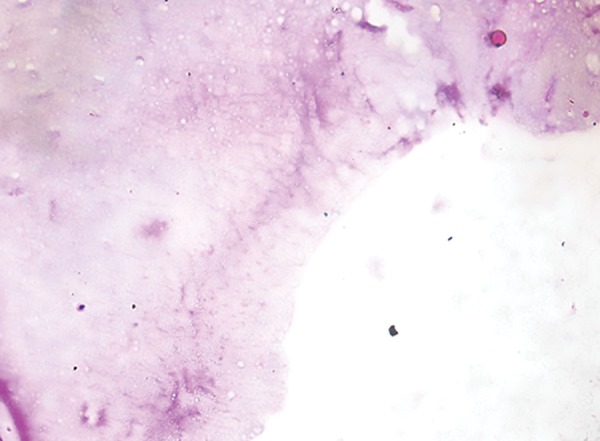
Representative histological section (40×) after treatment with Carie-Care showing slight amount of dentinal tubule destruction and less bacterial remnants in the cavity floor

**Fig. 2:**
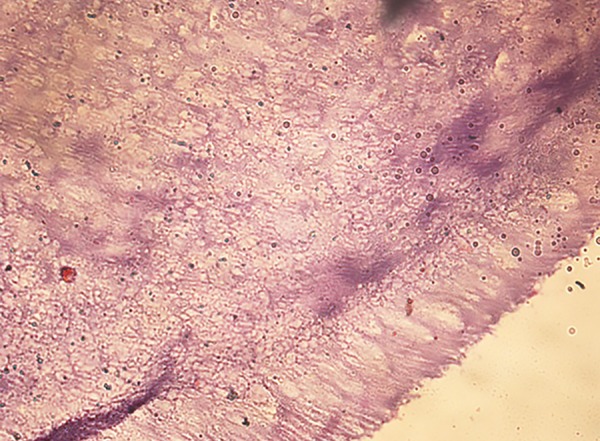
Representative histological section (40×) after treatment with Er:YAG laser showing irregular cavity floor and infected layer almost completely removed with no destruction of dentinal tubules

**Fig. 3:**
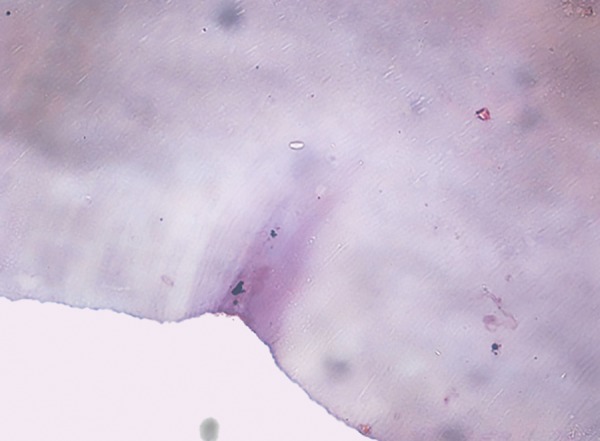
Representative histological section (40×) after treatment with round tungsten carbide bur showing smooth cavity floor, without irregularities and with marked dentinal tubule destruction

After caries excavation, the samples were decalcified with 10% hydrochloric acid at room temperature for 24 hours. After decalcification, the samples were dehydrated in ascending degrees of alcohol (70, 80, 90 and 100%). Dehydrated samples were cleared in xylene, embedded in paraffin, and then cut into 5 um sections. These samples were then stained with Schiff’s reagent for the evaluation of bacterial deposits using conventional light microscope (Figs 1 to 3).

Presence of bacteria among groups was scored according to Peric and Markovic^9^

- Score 0: No bacteria detected;
- Score 1: Bacteria in the cavity floor;
- Score 2: Bacteria penetrating dentin tubules up to one half of the distance between the cavity floor and the pulp chamber.

## EVALUATION OF MORPHOLOGICAL CHANGES

**Figs 4A and B:**
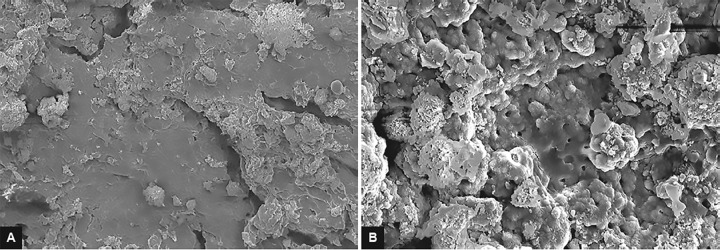
(A) Micrograph representative of specimen from group I (2,000×) showing irregularities on the cavity surface with minimally formed smear layer and few open dentinal tubules. (B) Micrograph representative of specimen from group I (5,000×) showing irregularities on the cavity surface with minimally formed smear layer and few open dentinal tubules

**Figs 5A and B:**
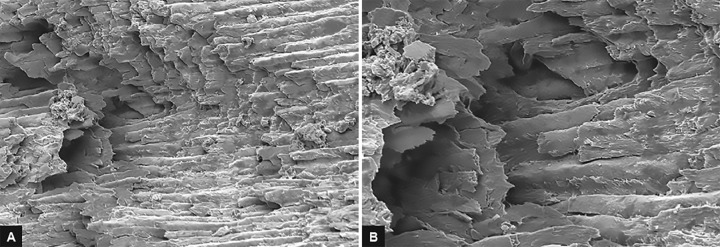
(A) Micrograph representative of specimen from group II (2,000×) showing irregular rugged appearance and no smear layer formation and opening of dentinal tubule orifices. (B) Micrograph representative of specimen from group II (5,000×) showing irregular rugged appearance and no smear layer formation and opening of dentinal tubule orifices

**Figs 6A and B:**
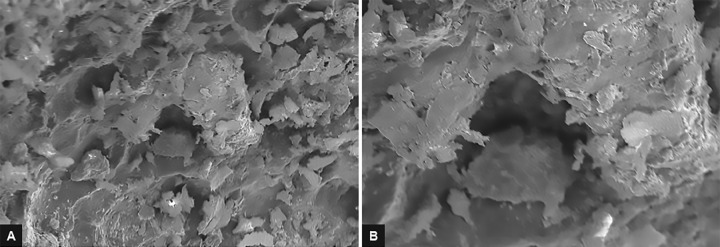
(A) Micrograph representative of specimen from group III (2,000×) showing rougher cavity floor with well-defined smear layer and occluded dentinal tubules. (B) Micrograph representative of specimen from group III (5,000×) showing rougher cavity floor with well-defined smear layer and occluded dentinal tubules

After excavation of caries, only representative samples from the above three groups were prepared for SEM evaluation. These samples were kept in 4% glutaralde-hyde solution for 1 hour at room temperature and then were rinsed with distilled water. Samples were then immersed in sodium cacodylate for 90 min followed by dehydration in the ascending grades of ethanol for 1 hour and then were dried. These dried samples were mounted on a metal stand, gold sputter coated, and were examined under SEM. Images at 2,000× and 5,000× magnification were obtained. Each SEM photomicrograph was evaluated and morphological changes were described^7^ (Figs 4 to 6).

### Statistical Analysis

Statistical analysis was carried out using Statistical Package for the Social Sciences, version 16. Results were subjected to statistical analysis using Kruskal–Wallis test and Mann-Whitney U test for intergroup comparisons (p-value <0.001) and further subjected to Fisher’s exact test for distribution of scoring.

## RESULTS

### Light Microscopy

**Graph 1:**
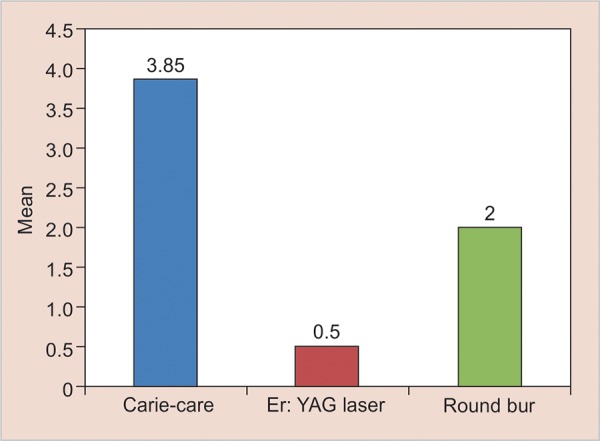
Intergroup comparison of number of bacterial counts after caries excavation in all the three groups

Based on light microscopic observations, least bacterial deposits were observed in group II, Er:YAG laser followed by group III, round tungsten carbide bur. Bacterial deposits were observed to be more in group I, Carie-Care. Therefore, the difference in the number of bacterial deposits was significant among all the groups (p < 0.001) (Table 1 and Graph 1).

On pairwise comparison, it was found that group I showed statistically significant results in comparison with groups II and III. Group II showed statistically significant results in comparison with group III. Also Kruskal–Wallis test showed that there was a statistically significant difference among all the three groups (p-value < 0.001) (Table 1).

**Graph 2:**
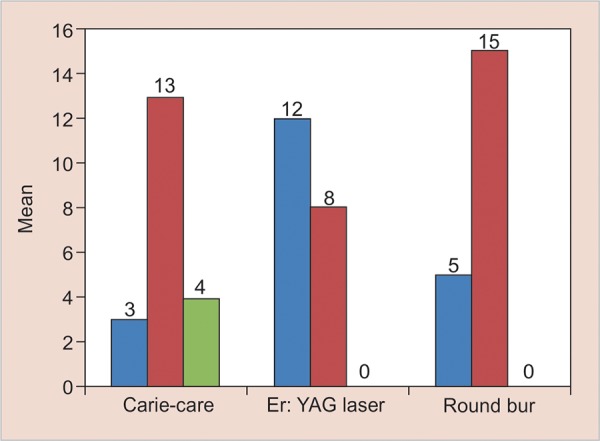
Distribution of scoring given to caries excavated dentin in all the three groups

Statistically significant difference was present regarding presence of the bacteria in cavity floor and bacteria penetrating varying depths of the dentinal tubules among the groups (p-value: 0.003, Fisher’s exact test). 60% of the samples in Er:YAG laser group were bacteria-free, while 25 and 15% of samples were bacteria-free in round bur and Carie-Care groups respectively (Table 2 and Graph 2).

**Table 1:** **Table 1:** Descriptive statistics showing intergroup comparison of number of bacterial counts following caries excavation in all the three groups using Kruskal–Wallis test and Mann-Whitney U test

										*Kruskal–Wallis test*				*Mann-Whitney U test (p-value)*					
*Group*		*N*		*Mean (SD)*		*Range*		*Median (Q1-Q3)*		*Chi-square value*		*p-value*		*I vs II*		*I vs III*		*II vs III*	
Carie-Care		20		3.85 (2.62)		0-10		3.50 (2-5.75)		22.88		<0.001*		<0.001*		0.02*		0.001*	
Er:YAG laser		20		0.50 (0.69)		0-2		0 (0-1)											
Round bur		20		2.00 (1.52)		0-5		2 (0.25-3)											

*p < 0.05 statistically significant; p > 0.05, nonsignificant; SD: Standard deviation

**Table 2:** **Table 2:** Descriptive statistics showing distribution of scoring after caries excavation in all the three groups^9^

		*Score*								*Fisher’s exact test*	
*Group*		*0*		*1*		*2*		*Total*		*p-value*	
Carie-Care		3 (15.0%)		13 (65.0%)		4 (20.0%)		20		0.003*	
Er:YAG laser		12 (60.0%)		8 (40.0%)		0		20			
Round bur		5 (25.0%)		15 (75.0%)		0		20			
Total		20 (33.3%)		36 (60.0%)		4 (6.7%)		60			

*p < 0.05 statistically significant; p > 0.05, nonsignificant

## DISCUSSION

Treatment of carious primary teeth poses a challenge to pediatric dentists. The scenario becomes all the more complicated when pulp is involved. Therefore, it is important for clinicians to treat the carious lesions as early as possible with maximum conservation of tooth structure, since primary teeth maintain the space for eruption of succedaneous teeth, preserve esthetics, prevent detrimental habits, and to maintain function.^10^

A recently introduced concept in chemomechanical caries removal has been brought to fruition by the introduction of Carie-Care which is available naturally, simple to use antimicrobial removing only infected dentin, and is cost-effective.^11^

Yet another minimally invasive method of caries removal came into existence with the introduction of lasers. Among these, Er:YAG laser is pointed out as the most promising tool because it can more effectively ablate enamel and dentin due to their highly efficient absorption power in both water and hydroxyapatite.^12^ Together with this, the added advantage of its minimally invasive nature, inherent safety, and reduced use of local anesthesia make Er:YAG laser a more attractive preposition for use in pediatric dentistry.^10^ Keeping these concepts in mind, Carie-Care and Er:YAG laser were chosen in this study and were compared with round tungsten carbide bur, which is a most widely used method of caries excavation in dentistry and is considered as gold standard.

The aim of modern concept of treatment of dentin caries is to remove not only the outer, permanently damaged “infected” layer of carious dentin, but also preserve the demineralized “affected” dentin which can be healed. In addition, depth of microbial invasion cannot be diagnosed with clinical criteria for complete caries removal.^13^ Therefore, the efficacy of caries excavation was assessed in terms of number of bacterial deposits using conventional light microscope.

Further, to substantiate the observations, SEM was used in the study to evaluate the morphological changes in caries-excavated dentin. The usage of SEM technology allows visualization of samples with different magnitudes without altering the focus. In addition, since SEM figures are in gray scale, the color of dentin does not influence in obtaining a correct focus, a limitation which is found in optical stereomicroscopes.^14^

In Carie-Care group, we observed that dentin surfaces showed irregularities with the presence of bacterial deposits, few openings of dentinal tubules, and minimal formation of smear layer. This finding in our study was in accordance with study conducted by Avinash et al who stated that the absence of a smear layer is a result of the specific preparation technique without thermal or mechanical effects and high pH.^13,15^

In our study, Er: YAG laser-ablated carious dentin showed scaly, irregular surface with rugged appearance with no smear layer formation. Dentin tubule orifices were open without any widening which reflects the explosive process of tooth structure removal. Additionally, peritubular dentin protruded slightly more from surrounding intertubular dentin. The highly irregular surface of the cavity floor without smear layer will provide a suitable surface for strong bonding with the composite materials. It may also provide an etching behavior which is not seen with the mechanical or chemomechanical system.^16^

In samples wherein caries was excavated by round tungsten carbide bur, we observed that the dentin surfaces showed bacterial deposits, well-formed smear layer, and occluded dentinal tubules. This typical feature of the drilled cavity structure, i.e., loosely attached debris-like smear layer, interferes with adhesion, wetting, penetration, and hardness of the prepared cavity.^17^

We observed in our study that bacterial deposits after caries excavation with Carie-Care were found to be high compared with that of round tungsten carbide bur. The efficacy of removing caries with round tungsten carbide bur is highest because it tends to over-prepare the cavities due to lack of sensitivity of tactile feedback. This results in gross removal of sound tooth structure with reduced control over the whole process.^18,19^

While in Carie-Care group, bacterial deposits was found to be more, as the extent of excavation is dependent on the operator’s decision, less extensive preparation, and presence of bacteria by the absence of smear layer in the chemomechanically treated cavities, which enables direct pushing of bacteria into the dentin tubules with hand instruments compared with the one with rotary instruments.^9^

We observed that least amount of bacterial deposits was in caries excavated with Er:YAG laser. This finding was due to the bactericidal effect of laser system on dentin surface.^20^ Having the possibility to ablate small areas with the Er:YAG laser helps reaching one of the most important goals of minimum intervention dentistry (MID), i.e., the maximum preservation of dental tissue. These results are probably attributable to the different ablation mechanism of Er:YAG laser resulting in a photoacoustic effect. Laser irradiation was performed in the noncontact mode, in the presence of a constant water flow during laser exposure. Also, water irrigation seems to effectively prevent thermal damage. The spray allows cleaning of the ablation site, supplies an increased efficiency for the ablation rate, and promotes the ablation process.^21^

In the modern era, the concept of “extension for prevention” has greatly been replaced by the concept of “prevention of extension” which ensures a maximum preservation of tooth structure destruction. Thus, from the present study, it is clear that caries excavation using Er:YAG laser and Carie-Care in primary teeth offers a number of advantages for the child patient in terms of minimally invasive dentistry concepts.

## CONCLUSION

From the present study, it can be concluded that among the three different methods of caries excavation, Er:YAG laser was found to be more effective and along with Carie-Care, it was consistent with minimal invasive preparation providing clean surfaces and strong microretentions, ideal for adhesive restorations.

## CLINICAL SIGNIFICANCE

Laser-induced caries excavation by Er:YAG laser and chemomechanical method of caries removal by Carie-Care can be considered as future of noninvasive pediatric and preventive dentistry.
